# Migraine and the Excessive Dispensation of Triptans: A Real-World Evidence Study of Colombian Patients

**DOI:** 10.31083/RN46355

**Published:** 2026-01-26

**Authors:** Luis Fernando Valladales-Restrepo, Catalina Díaz-Arteaga, Luis Fernando Navarrete-Santa, Melba Jasbleidy Santander-Pai, José Manuel Zapata-Orozco, Guisela Villa-Arroyave, Jorge Enrique Machado-Alba

**Affiliations:** ^1^Grupo de Investigación en Farmacoepidemiología y Farmacovigilancia, Universidad Tecnológica de Pereira-Audifarma S.A, 660003 Pereira, Risaralda, Colombia; ^2^Grupo de Investigación Biomedicina, Facultad de Medicina, Fundación Universitaria Autónoma de las Américas, Institucion Universitaria Visión de las Américas, 660004 Pereira, Risaralda, Colombia; ^3^Semillero de Investigación en Farmacología Geriátrica, Grupo de Investigación Biomedicina, Facultad de Medicina, Fundación Universitaria Autónoma de las Américas, Institucion Universitaria Visión de las Américas, 660004 Pereira, Risaralda, Colombia

**Keywords:** migraine, chronic headache, medication overuse headache, triptans, inappropriate prescribing, pharmacoepidemiology

## Abstract

**Background:**

The aim of this study was to determine the use and safety of triptans in a group of patients with migraine who had excessive dispensings of antimigraine drugs.

**Methods:**

This was a cross-sectional study of patients with excessive dispensings of triptans identified by a pharmaceutical manager. The DrugBank database was used to determine rational amounts of triptans.

**Results:**

A total of 9147 patients used triptans, 44.6% of whom received excessive dispensings. A sample of 355 patients was selected, 22.8% of whom received regular doses of triptans daily. Adverse events were common (41.1%), and some patients experienced chronic headache (32.4%) and medication-overuse headache (MOH) (8.2%). Increasing age [adjusted odds ratio (aOR): 1.042; 95% confidence interval (CI): 1.008–1.077], a history of migraine for more than 10 years (aOR: 3.73; 95% CI: 1.37–10.16), previous dispensings of simple analgesics (aOR: 2.463; 95% CI: 1.001–6.057), and concomitant psychiatric illnesses (aOR: 3.583; 95% CI: 1.452–8.844) were associated with a higher probability of MOH.

**Conclusions:**

In this study conducted in a middle- to low-income Latin American country, triptans were commonly dispensed for patients with migraine, and their dosage did not comply with the recommendations of clinical practice guidelines for some patients. Increasing age, history of migraine ≥10 years, previous use of simple analgesics, and the presence of concomitant psychiatric disorders were associated with a higher probability of MOH. These findings reflect prescribing and dispensing patterns within the studied health-care context and may not fully represent the use of over-the-counter triptans or practices in other settings.

## 1. Introduction

Migraine is one of the most common neurological diseases, affecting more than 1100 million people worldwide [1]. Migraine is the most common type of headache after tension headache, but it has the highest morbidity and disability rates [2, 3, 4]. The prevalence of migraine is 14.0% globally [5], 17.0% in Latin America [5], and 19.6% in Colombia [6]. The acute management of migraine involves simple analgesics such as nonsteroidal anti-inflammatory drugs (NSAIDs) or acetaminophen and specific medications such as triptans [4, 7, 8]. However, a significant proportion of people require prophylactic interventions with β-blockers and antiepileptic drugs, among others, due to very frequent headache attacks or the inability to control headache with acute pharmacological therapy [4, 7, 8].

The frequent use of medications to treat acute headaches can lead to an increase in the frequency of pain episodes and a transition from episodic headache to chronic headache [9, 10, 11]. Medication-overuse headache (MOH) is a chronic type of headache that is associated with the excessive use of medications such as triptans, ergot derivatives, NSAIDs, acetaminophen, or opioids [9, 10, 11, 12]. There are more than 58.5 million people with MOH worldwide [3]. MOH can involve any type of headache, but the most common type involves the chronicity and transformation of migraine [13]. Compared with other analgesic therapies, the excessive use of triptans causes MOH to develop more quickly [13].

The negative impact of MOH on the quality of life of affected individuals is greater than that of episodic migraine, as is the cost of care for MOH [9, 13]. The excessive or inappropriate use of analgesics in individuals with headache attacks is one of the most important modifiable risk factors [11, 14]. Therefore, it is crucial to prevent the development of MOH by identifying patients who are at greater risk, such as those who have excessive dispensings of antimigraine drugs, such as triptans [13]. Furthermore, the prevalence of MOH in patients receiving triptans ranges from 2.1% to 46.3% [15].

The Colombian healthcare system provides coverage to the entire population through two main regimes (contributory and subsidized regimes). All employees, pensioners, and self-employed workers must be enrolled in the contributory regime. Coverage for individuals who are unable to pay is provided by the subsidized regime, which is financed by the state. The health benefits plan is the same for both regimes and includes some triptans in different pharmaceutical forms for the management of migraine attacks [16]. In Colombia, some pharmaco-epidemiological studies have been carried out in patients with migraine, but there are no reports that address the excessive dispensing of triptans [17, 18, 19]. The objective of this study was to determine the use profile and safety of triptans in a group of patients with migraine who had excessive dispensings of antimigraine drugs in Colombia.

## 2. Patients and Methods

### 2.1 Study Design and Patients

A cross-sectional study of patients receiving excessive dispensings of triptans for the management of migraine was carried out. The patients were identified from the medication dispensing database of a pharmaceutical management system, which includes 9.5 million people who are enrolled in the Colombian health system, representing 16.3% of the Colombian population. The patients are distributed across all regions of Colombia and account for 30% of the population enrolled in the contributory regime and 6% of the population enrolled in the subsidized regime. The study followed the Strengthening the Reporting of Observational Studies in Epidemiology (STROBE) guidelines (**Supplementary Material-STROBE-checklist**).

**Fig. 1.**
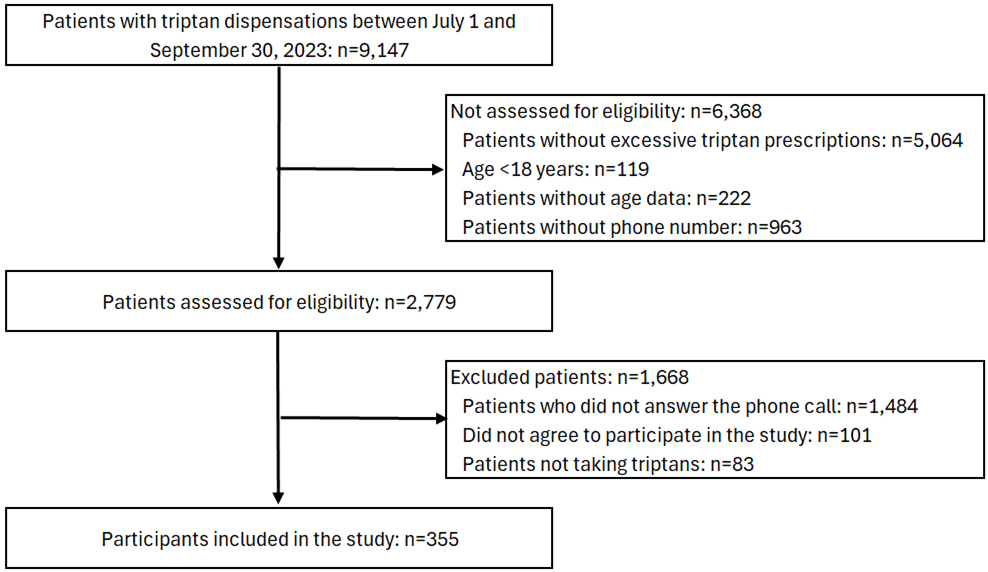
**Study flow diagram with patients between July 1 and September 30, 2023**. Participants included in the study (n = 355) were selected randomly.

Male or female patients aged ≥18 years who were from any city of origin and had excessive dispensings of oral or nasal triptans between July 1 and September 30, 2023, were selected (Fig. 1). The first dispensing of triptan was considered the index date for each patient. Patients with excessive dispensings of triptans were identified, considering what was reported in DrugBank https://go.drugbank.com/ [20]. DrugBank is a free-access online database that contains extensive biochemical and pharmacological information on drugs, their mechanisms, and their treatment targets [21]. This database is widely used in biomedical research, with an average of more than 30 million visits per year [21]. DrugBank considers the treatment of 5 headaches within a 30-day period with naproxen/sumatriptan, 4 headaches within a 30-day period with sumatriptan or naratriptan, and 3 headaches within a 30-day period with zolmitriptan or eletriptan to be safe [20]. Therefore, exceeding the following units of triptan per month was considered excessive dispensing of the medication in this study (**Supplementary Table 1**) [20]:

- Naproxen/sumatriptan: 500/85 mg tablets at >12 tablets/month.
- Sumatriptan: 50 mg tablets at >16 tablets/month, 100 mg tablets at >8 tablets/month, an 80 mg/mL oral solution/10 mL >1 bottle/month, and a 20 mg/dose nasal solution/6 doses at >2 nasal sprays/month.
- Naratriptan: 2.5 mg tablets at >7 tablets/month.
- Eletriptan: 40 mg tablets at >12 tablets/month.
- Zolmitriptan: 2.5 mg tablets at >6 tablets/month and a nasal solution 5 mg/dose/7 doses at >1 nasal spray/month.

These thresholds were used because DrugBank provides precise information on the maximum recommended doses for each triptan. Since triptans are intended for acute migraine treatment, these dosing parameters allow dispensed units to be translated into estimated potential doses and enable the definition of a threshold even without direct data on medication intake. In this study, these values were adopted as an operational, reproducible criterion to identify dispensed quantities that exceed what would be therapeutically expected. The recommendations of DrugBank are easy to implement in everyday medical practice compared with the defined daily doses methodology for determining excessive medication dispensing [20, 21, 22].

A total of 2779 people who met the inclusion criteria and had telephone contact information were identified (Fig. 1). A total stratified random sample of 337 people was calculated to estimate the proportion of patients with MOH using Epi Info software [Centers for Disease Control and Prevention (CDC), 2024. Epi Info, Version 7.2.5, Atlanta, GA, USA]. A confidence interval of 95%, an error rate of 5% and an expected frequency of 50% were considered [23]. The selected patients were contacted by telephone to inquire about the clinical characteristics of their migraines, complications, triptan doses, adverse events and clinical response to the drug. All patients provided verbal consent prior to completing the interview. Patients who could not be contacted by telephone on three different occasions, those who were prescribed triptans but did not take them, those who refused to participate in the study, and those without a diagnosis of migraine were excluded (Fig. 1).

### 2.2 Variables

A database with the following variables was designed using the information obtained directly from the patient (with prior verbal informed consent) and from the pharmaceutical manager (Audifarma SA):

(a) Sociodemographic characteristics: age, sex, regime type (contributory or subsidized), city of origin, education level, occupation type, and marital status. The city of origin was categorized by department according to the region of Colombia as follows, considering the classification of the National Administrative Department of Statistics (DANE) of Colombia: Bogotá-Cundinamarca, Caribbean, Central, Pacific, and Eastern-Orinoquia-Amazoniaía.

(b) Clinical characteristics:

- Characteristics of migraine: periodicity (average number of attacks per month), intensity (severity) according to the numerical verbal scale (1–3 points: mild pain; 4–6 points: moderate pain; and 7–10 points: severe pain), and evolution of the pathology (in years). Cases of chronic migraine and MOH were identified according to the criteria of the International Classification of Headache Disorders, third edition (ICHD-3) [10]. People who required management in the emergency department in the last 3 months due to the intensity of their headache attack and those who had a specialized neurology evaluation in the last year were identified.
- Chronic comorbidities in the last 12 months from the index date were identified using International Classification of Diseases, version 10 (ICD-10) codes.

(c) Pharmacological characteristics:

- Triptan: type of initial prescriber (e.g., general, neurologist, or internist), type of medication (e.g., eletriptan, naproxen/sumatriptan, naratriptan, sumatriptan, or zolmitriptan), pharmaceutical form (tablet, oral solution, or nasal solution), and dosage. The pattern of medication use was assessed as either intake during migraine attacks or regular daily intake (e.g., once a day, twice a day), and the number of dispensings of triptans in the last year from the index date. Data on the occurrence of adverse events (e.g., emesis, drowsiness, myalgia, dizziness), the absence of pain 24 hours after the triptan was administered (always, almost always, sometimes, almost never, never), the intake or non-intake of all dispensed medication units, and the reasons for suspending the therapy were collected.
- Other analgesics or antiemetics include acetaminophen, NSAIDs (e.g., diclofenac, naproxen, and ibuprofen), ergot derivatives (ergotamine), opioids (e.g., tramadol, codeine, and oxycodone), and antiemetics (e.g., metoclopramide and domperidone).
- Antimigraine prophylaxis included β-blockers (bisoprolol, propranolol, metoprolol), antiepileptics (topiramate, valproic acid), tricyclic antidepressants (amitriptyline, imipramine), angiotensin II receptor antagonists (candesartan), calcium antagonists (flunarizine), and calcitonin gene-related peptide (CGRP) receptor antagonists (galcanezumab, erenumab). The use of antimigraine drugs in the last 12 months from the index date was considered antimigraine prophylaxis. The use of β-blockers, antiepileptics, tricyclic antidepressants, angiotensin II receptor antagonists, or calcium antagonists was considered conventional prophylaxis.
- Other medications were grouped into the following categories: antiulcer drugs, lipid-lowering drugs, antihistamines, antidiabetics, antihypertensives, and antidepressants.

### 2.3 Statistical Analysis

The data were analyzed with the Statistical Package for the Social Sciences (SPSS), version 26.0 for Windows (IBM Corp., Armonk, NY, USA). Data on categorical variables are presented as frequencies and percentages. The quantitative variables did not follow a normal distribution according to the Kolmogorov–Smirnov test; therefore, the data are presented as medians and interquartile ranges. Bivariate analysis was performed using the *X*^2^ test or Fisher’s exact test and the Mann-Whitney U test. To assess the representativeness of the study sample, some variables (sex, age, geographic region, type of health insurance, and type of triptan) were compared between participants and non-participants.

An exploratory binary logistic regression was performed. The dependent variable was whether the diagnostic criteria for MOH were met (yes/no). The independent variables (covariates) included in the model were those that showed statistical significance in the bivariate analysis or those that demonstrated biological plausibility (e.g., sex, age, geographic region, characteristics of the disease, and pharmacological history). The intro method was used to select the variables, with careful assessment of collinearity among covariates. The crude and adjusted odds ratio (OR) are shown with its 95% confidence interval (CI). Statistical significance was considered at *p* < 0.05. The performance of the logistic regression model was evaluated using several statistical procedures. Multicollinearity among the predictors was assessed through tolerance values and the Variance Inflation Factor (VIF). Model calibration was examined using the Hosmer–Lemeshow goodness-of-fit test and visually inspected through a calibration plot comparing predicted and observed probabilities. The model’s discriminative ability was determined by estimating the area under the receiver operating characteristic (ROC) curve.

## 3. Results

Between July and September 2023, 9147 patients were identified as having been dispensed triptans. Among these patients, 44.6% (n = 4083) were considered to have excessive dispensings (Fig. 1). **Supplementary Table 2** compares participants (n = 355) with non-participants (n = 2424). Overall, the groups were similar across most variables. Participants were slightly younger (median 40.0 vs. 42.0 years) and differed in the distribution of some geographic regions and health insurance categories. No significant differences were observed in sex distribution and triptan type.

### 3.1 Sociodemographic Characteristics

In total, 355 patients were selected from 81 different cities. A total of 85.9% (n = 305) of the participants were women, and the median age was 40.0 years (29.0–51.0 years). A total of 49.6% (n = 176) of the patients were <40 years old, 45.1% (n = 160) were between 40 and 64 years old, and 5.4% (n = 19) were ≥65 years old. The majority of the patients were from the Caribbean region, were married or in common-law relationships, had secondary schooling, carried out household activities, and were affiliated with the contributory regime of the health system (Table 1).

**Table 1.** **Sociodemographic and clinical variables of a group of patients with migraine and excessive triptan dispensing, Colombia**.

Variables			Total	
			n = 355	%
Sociodemographic			-	
	Women		305	85.9
	Age, median (interquartile range)		40.0 (29.0–51.0)	
	Origin		-	-
		Caribbean Region	124	34.9
		Bogota-Cundinamarca Region	88	24.8
		Central Region	81	22.8
		Pacific Region	45	12.7
		Eastern-Orinoquia-Amazonia Region	17	4.8
	Civil status		-	-
		Married or in common-law relationships	200	56.4
		Single	135	38.0
		Separated or widowed	20	5.6
	Education level		-	-
		Primary	54	15.2
		Secondary	166	46.8
		University	134	37.7
		Missing	1	0.0
	Occupation		-	-
		Home activities	98	27.6
		Independent	44	12.4
		Student	19	5.4
		Nursing	16	4.5
		Pensioner	12	3.4
		Others	166	46.8
	Contributory regime		257	72.4
	Subsidized regime		98	27.6
Migraine			-	-
	Type of migraine		-	-
		Without aura	208	58.6
		With aura	147	41.4
	Periodicity of crises		-	-
		<5 headache episodes per month	130	36.6
		5–14 headache episodes per month	145	40.8
		≥15 headache episodes per month	80	22.5
	Crisis intensity		-	-
		Severe	318	89.6
		Moderate	31	8.7
		Mild	5	1.4
		Missing	1	0.0
Comorbidities			-	-
	Endocrine		127	35.8
	Cardiovascular		105	29.6
	Digestive		59	16.6
	Psychiatric		55	15.9
	Rheumatological		52	14.6
	Neurological		31	8.7
	Others		74	20.8

### 3.2 Clinical Characteristics

The median duration since the diagnosis of migraine was 7.0 years (interquartile range: 2.0–16 years). The patients usually had migraine without aura (58.6%), with five or more attacks per month (63.3%), and severe intensity (89.6%) (Table 1). A total of 32.4% (n = 115) of the patients had chronic headache, and 8.2% (n = 29) had MOH. In the last 3 months, 23.1% (n = 82) of the patients had to visit the emergency department because of the intensity of their headaches. A total of 62.5% (n = 222) of the participants were assessed by neurology in the previous year. The most frequent comorbidities were hypertension (n = 103, 29.0%), anxiety disorders (n = 50, 14.1%), irritable bowel syndrome (n = 47, 13.2%), hypothyroidism (n = 46, 13.0%), dyslipidemia (n = 43, 12.1%), and diabetes mellitus (n = 42, 11.8%).

### 3.3 Pharmacological Characteristics

The most commonly used triptan was sumatriptan (Table 2), and tablets were the most commonly used pharmaceutical form (n = 347; 97.7%). A total of 50.7% (n = 180) of the patients had received triptans in the previous year (median 1.0 dispensings; interquartile range: 0–2 dispensings). Of these dispensings, 74.7% (n = 399/534) were excessive. Seventeen different ways of using triptans were identified; 77.2% (n = 274) involved the use of triptans in headache attack schemes, and 22.8% (n = 81) involved regular doses every day (Table 2). A total of 50.1% (n = 178) of the patients reported that they always or almost always experienced an absence of pain 24 hours after the triptan was administered (Table 2). A total of 41.1% (n = 146) of the patients reported having any adverse events, especially dizziness/vertigo, nausea, and drowsiness (Table 2). A total of 42.0% (n = 149) of the patients did not use all the triptan units that had been dispensed. A total of 23.1% (n = 82) of the patients discontinued triptan treatment due mainly to adverse events (n = 25/82; 30.5%); the patients complied with the triptan treatment regimen and had no other medication orders to file with the pharmacy (n = 21; 25.6%) or lacked clinical improvement (n = 10; 12.2%).

**Table 2.** **Pharmacological variables of a group of patients with migraine and excessive triptan dispensing, Colombia**.

Variables		Total	
		n = 355	%
Initial prescribing physician		-	-
	Neurology	205	57.7
	General practitioner	107	30.1
	Internist	37	10.4
	Others	6	1.7
Triptan type		-	-
	Sumatriptan	129	36.3
	Naratriptan	124	34.9
	Sumatriptan/naproxen	98	27.6
	Eletriptan	4	1.1
Posology		-	-
	1 dose in the crisis	152	42.8
	1 dose in the crisis, if there is no improvement, repeat after 2 hours	68	19.2
	1 dose each day	55	15.5
	1 dose in the crisis, if there is no improvement, repeat after 4 hours	21	5.9
	1 dose in the crisis, if there is no improvement, repeat after 6 hours	14	3.9
No pain after 24 hours		-	-
	Always	102	28.7
	Almost always	76	21.4
	Sometimes	95	26.8
	Almost never	15	4.2
	Never	67	18.9
Any adverse event		146	41.1
	Dizziness/Vertigo	55	15.5
	Nausea	46	13.0
	Drowsiness	46	13.0
	Epigastric pain	36	10.1
	Diaphoresis	16	4.5
Concomitant use of analgesics and/or antiemetics		-	-
	Non-steroidal anti-inflammatory drugs	118	33.2
	Acetaminophen	92	25.9
	Opioids	36	10.1
	Metoclopramide	17	4.8
	Domperidone	3	0.8
Anti-migraine prophylaxis		-	-
	Antiepileptics	99	27.9
	β-blockers	68	19.2
	Flunarizine	68	19.2
	Tricyclic antidepressants	53	14.9
	Botulinum toxin	37	10.4
	Calcitonin Gene Related Peptide receptor antagonists	12	3.4

A total of 44.8% (n = 159) of the patients used other analgesics, predominantly NSAIDs, in the last 3 months (Table 2). A total of 59.7% (n = 212) of the patients received antimigraine prophylaxis, mainly with conventional drugs (n = 202; 56.9%), with topiramate (n = 72; 20.3%) and flunarizine predominating (n = 68; 19.2%) (Table 2). Overall, 22.0% (n = 78) of the patients had ergotamine dispensings in the past year. The main medications to other morbidities used were antiulcer drugs (n = 197, 55.6%), antihistamines (n = 171, 48.2%), antidepressants (n = 140, 39.4%), lipid-lowering drugs (n = 115, 32.4%), and antihypertensives and diuretics (n = 103; 29.0%).

### 3.4 Multivariate Analysis

The bivariate analysis can be observed in **Supplementary Table 3**. Binary logistic regression revealed that increasing age, patients who experienced migraine for more than 10 years, patients who were previously use of simple analgesics, patients who received conventional prophylaxis, and patients who had concomitant psychiatric disorders were all associated with a higher probability of MOH (Table 3). The logistic regression model satisfied all required statistical assumptions. The tolerance values (0.817–0.983) and Variance Inflation Factors (1.018–1.223) indicated the absence of multicollinearity among the explanatory variables. The Hosmer–Lemeshow test (*p* = 0.991) demonstrated good model fit, and the calibration plot showed an appropriate agreement between the predicted probabilities and the observed proportions (**Supplementary Fig. 1**). The model’s discriminatory ability was good, as evidenced by an area under the ROC curve of 0.861 (95% CI: 0.798–0.924) (**Supplementary Fig. 2**). Overall, the model exhibited strong statistical performance.

**Table 3.** **Binary logistic regression of variables associated with a higher probability of Medication-Overuse Headache in patients with migraine and excessive triptan dispensing, Colombia**.

Variables	Crude OR	95% CI		*p*-value	Adjusted OR	95% CI		*p*-value
		Lower	Upper			Lower	Upper	
Woman (yes/no)	0.769	0.279	2.118	0.611	0.420	0.121	1.456	0.171
Age (continuous)	1.060	1.031	1.091	<0.001	1.042	1.008	1.077	0.015
Origin Bogota-Cundinamarca Region (yes/no)	1.673	0.747	3.750	0.211	1.875	0.722	4.864	0.196
History of migraine ≥10 years (yes/no)	4.800	1.993	11.560	<0.001	3.738	1.375	10.161	0.010
Sumatriptan (yes/no)	1.077	0.492	2.358	0.852	0.875	0.351	2.181	0.775
Correct dosage (yes/no)	0.515	0.233	1.136	0.100	0.538	0.218	1.331	0.180
Absence of pain after 24 hours -always or almost always- (yes/no)	1.246	0.581	2.673	0.572	1.271	0.526	3.076	0.594
Previous use of simple analgesics (yes/no)	2.990	1.321	6.766	0.009	2.463	1.001	6.057	0.050
Conventional prophylaxis (yes/no)	5.261	1.791	15.458	0.003	4.370	1.356	14.082	0.014
Concomitant psychiatric illnesses (yes/no)	4.646	2.076	10.397	<0.001	3.583	1.452	8.844	0.006

OR, Odds Ratio; CI, Confidence Interval.

## 4. Discussion

In this study, we investigated the sociodemographic and clinical characteristics of patients with migraine who received excessive dispensings of triptans, as well as the use of these medications, the most common adverse reactions, and the factors related to MOH. A total of 8.2% of the patients developed MOH, and several factors were associated with a higher probability of its occurrence, including older age, longer disease duration, prior use of analgesics, and the presence of psychiatric comorbidities. The World Health Organization announced that the inappropriate use of drugs is common worldwide. In addition, the excessive or improper use of drugs leads to wasted resources and widespread health risks [24]. By understanding how medications are used in the real world, strategies to improve physicians’ prescribing habits can be designed to increase medication efficacy and safety [25].

In this sample of patients with migraine, women and young adults predominated, as reported in other studies [15, 17, 19, 26, 27, 28, 29, 30, 31, 32, 33]. Similarly, migraine without aura prevailed, and the attacks were usually severe, which is consistent with the literature [26, 27, 28, 32]. Sumatriptan was the most commonly prescribed triptan, which is consistent with previous reports in the country [18, 19]. Zolmitriptan was previously found to predominate in Denmark and the Netherlands (52.0%–74.8%) [31, 34], while zolmitriptan predominated in Australia and France (32.5%–42.6%) [33, 35], and rizatriptan predominated in Japan (33.6%) [30]. These differences in drug use patterns may be due to various reasons, including the availability of drugs in each country, health system coverage, doctors’ medication use habits, academic training, and the clinical response to triptans [18, 19, 30, 31, 33, 34, 35]. With respect to effectiveness, half of the patients were always or almost always pain free within 24 hours of medication intake, which is consistent with the findings of a systematic review and meta-analysis of clinical trials [36]. Gepants are recommended for patients who do not respond satisfactorily to triptan therapy [37, 38]. They are effective and well tolerated, with a minimal risk of MOH [37, 38]. However, at the time the study was conducted (2023), these medications were not available in Colombia [16].

Among the triptan users, 44.6% had excessive dispensings, which is consistent with findings reported in Italy (43.3%) [32] and higher than those reported in other European countries (2.3%–21.5%) [31, 34, 35], Australia (6.0%) [33], and Japan (3.7%) [30]. Find *et al*. [39] conducted a multicenter study across several European countries (Denmark, Germany, Italy, and Spain) and two Latin American countries (Argentina and Chile), reporting that excessive triptan use was substantially more common in Europe than in Latin America (30.8% vs. 5.6%, respectively). However, the methodology of these investigations was different from that proposed in this report, so these findings may not be fully comparable [30, 31, 32, 33, 34, 35, 39]. Additionally, 22.8% of the patients used unapproved doses of triptans [20, 40]. The correct way to use triptans is by administering an initial dose at the time of the migraine attack, which can be repeated after 2 hours if there is no improvement in pain [20, 40]. Surprisingly, some patients used these drugs on a daily basis. These findings are consistent with those reported in a Colombian study that included patients with migraine who were treated with ergotamine [17]. The study revealed 26 different dosages, of which 99.5% were inappropriate; in 34.6% of the patients, ergotamine was used for indications that were not approved by regulatory agencies [17].

The adverse events that were documented in this investigation are consistent with those reported in other studies [20, 26, 40]. According to a meta-analysis of clinical trials, triptans were associated with a greater probability of adverse events than placebo or other pain relievers, such as acetaminophen and NSAIDs [26]. The risk increased when these drugs were used at high doses [26]. In addition, 8.2% of the patients experienced MOH, which is in line with a systematic review that included 20 studies conducted in Europe, North America, and Asia [15]. The prevalence of MOH among patients who received triptans ranged between 2.1% and 46.3% [15]. It is essential to limit the frequency of acute analgesic use to prevent MOH, as outlined in clinical guidelines, which recommend restricting triptan use to no more than nine days per month [10, 41]. Several strategies can be implemented to control excessive drug dispensing, such as controlling the number of units allowed per drug from health insurers, health providers, or pharmaceutical managers. The use of electronic drug management systems would allow alerts to be issued when physicians enter medication orders with excessively high quantities, enabling the physician to adjust the treatment regimen in a timely manner [42]. The use of electronic health systems reduces errors and increases patient safety [42, 43].

According to the literature, the frequent use of analgesics is a risk factor for the development of MOH [9, 10, 12, 14]. This finding is consistent with the previous use of other pain relievers in this study. It is important to implement continuing education programs on the correct use and dosage of medications that can improve the clinical practice of physicians [25, 44]. Migraine prophylaxis was also associated with a higher probability of MOH, which is similar to the Chronic Migraine Epidemiology and Outcomes (CaMEO) Study conducted in the United States [28]. However, this association should be interpreted with caution, as the cross-sectional design does not allow for causal inference. The finding may reflect confounding by indication: patients with more severe, frequent, or disabling migraine are more likely to be prescribed prophylactic therapy, and these same clinical characteristics also increase the likelihood of frequent use of abortive medication, thereby raising the risk of MOH [4, 7, 8]. Consequently, the observed association may reflect the underlying severity of the disorder rather than a true treatment effect. In addition, the presence of psychiatric comorbidities also increased the risk of MOH, which is consistent with other reports [27, 28, 29]. The dysfunction of neurotransmitters present in individuals with anxiety and depression disorders may play an important role in the development of MOH [11]. Other factors that increase risk, such as increased body mass index, smoking, admission to the emergency room in the last 6 months, severe migraine, and pain intensity, are mentioned in the literature [11, 27, 28, 29]; however, these factors were not found or were not evaluated in this study.

When interpreting the results, several limitations must be considered. First, the data were obtained from a group of patients insured by the Colombian health system, so the findings cannot be extrapolated to patients who do not have insurance. Second, although some differences were observed between participants and non-participants, the overall distribution of key variables was comparable, indicating that non-response or selection bias is unlikely to meaningfully influence the study’s conclusions. Third, the drug dispensing database does not allow the identification of drugs that patients may have purchased with their own resources. It also lacks information on complementary studies (e.g., neuroimaging studies), and it also does not allow differentiation between dispensing and medication intake. Fourth, recall biases may have occurred (e.g., in data on efficacy or adverse events) because the information was obtained by telephone. However, the calls were made the month after the dispensing. Fifth, a causal relationship between patient-reported adverse events and the use of triptans was not established, so it is possible that adverse events could be secondary to the use of other medications or be symptoms secondary to migraine. Sixth, the cross-sectional design of the study precludes the establishment of causality or the assessment of the temporal sequence between exposure and outcome. Because both variables are measured at a single point in time, it is not possible to determine whether the exposure preceded the outcome or whether the outcome may have influenced the exposure. Consequently, the findings should be interpreted strictly as statistical associations rather than causal relationships. However, the study included a significant number of people distributed across different geographic regions of Colombia, involving both men and women of different age ranges who were affiliated with the country’s health system.

## 5. Conclusions

Based on these findings, we conclude that excessive dispensing of triptans is common among migraine patients included in this study, predominantly women, with a mean age of 40.9 years and a disease duration of 11 years. These patients frequently present high blood pressure, anxiety disorders, and irritable bowel syndrome. In a considerable proportion of patients, the dosage does not correspond to the recommendation given by the clinical practice guidelines. More than 41% of the patients experienced adverse events, and 23% had to suspend treatment for this reason. Increasing age, history of migraine ≥10 years, previous use of simple analgesics, and the presence of concomitant psychiatric disorders were associated with a higher probability of MOH. However, these findings reflect only the prescriptions captured in our database and may not fully represent the use of over-the-counter triptans. Continuing education programs should be implemented, and more electronic drug management systems should be used to improve patient safety.

## Availability of Data and Materials

The daata used in this study are available on protocols (DOI: https://www.protocols.io/private/A2C3FF6834AB11EFA81B0A58A9FEAC02).

## Supplementary Material

Supplementary material associated with this article can be found, in the online version, at https://doi.org/10.31083/RN46355.
