# Supplementary material for: Migraine and the Excessive Dispensation of Triptans: A Real-World Evidence Study of Colombian Patients
Source: Rev Neurol. 2026 Jan 26;81(1):46355. doi: 10.31083/RN46355 (PMC12873677; doi:10.31083/RN46355)
Supplement: Supplementary file 1 [file 1576-6578-81-1-46355-s1.zip › Supplementary Material.docx]

**Supplementary Table**

**Supplementary Table 1**. Dosage forms, dose, maximum dose and number of episodes of safe use of triptans available in Colombia

| **Drugs** | **Dosage forms** | **Dose** | **Maximum dose** | **Safe** | **Rational/month** |
| --- | --- | --- | --- | --- | --- |
| Sumatriptan | Tablet 50 and tablet 100 mg | Initial dose: 50 to 100 mg. A second dose can be repeated at least 2 hours after the first | 200 mg/day | Its safety has not been established to treat more than 4 headache episodes in a 30-day period. | 16 and 8 |
|  | Oral solution 80 mg/mL/10 mL |  |  |  | 1 |
|  | Nasal solution 20 mg/dose/6 doses | Initial dose: 20 mg. A second dose can be repeated at least 2 hours after the first | 40 mg/day |  | 2 |
| Naproxen/Sumatriptan | Tablet 500/85 mg | Initial dose 500/85 mg. A second dose can be repeated at least 2 hours after the first | 1000/170 mg/day | Its safety has not been established for treating more than 5 headache episodes in a 30-day period. | 12 |
| Zolmitriptan | Tablet 2.5 mg | Initial dose: 2.5 mg. A second dose can be repeated at least 2 hours after the first | 5 mg/day | Its safety has not been established to treat more than 3 headache episodes in a 30-day period. | 6 |
|  | Nasal solution 5 mg/dose/7 doses | Initial dose: 5 mg. A second dose can be repeated at least 2 hours after the first | 10 mg/day |  | 1 |
| Naratriptan | Tablet 2.5 mg | Initial dose: 2.5 mg. A second dose can be repeated at least 4 hours after the first | 5 mg/day | Its safety has not been established to treat more than 4 headache episodes in a 30-day period. | 7 |
| Eletriptan | Tablet 40 mg | Initial dose: 40 mg. A second dose can be repeated at least 2 hours after the first | 80 mg/day | Its safety has not been established to treat more than 3 headache episodes in a 30-day period. | 12 |

**Supplementary Table 2**. Comparison of some variables between responders and non-responders.

| **Variables** | **Respondents** | | **Non-respondents** | | **p** |
| --- | --- | --- | --- | --- | --- |
|  | **n=355** | **%** | **n=2424** | **%** |  |
| Women | 305 | 85.9 | 2092 | 86.3 | 0.628 |
| Age, median (interquartile range) | 40.0 (29.0-51.0) | | 42.0 (32.0-52.0) | | 0.027 |
| Origin | - | - | - | - | - |
| Caribbean Region | 124 | 34.9 | 638 | 26.3 | 0.001 |
| Bogotá-Cundinamarca Region | 88 | 24.8 | 845 | 34.9 | <0.001 |
| Central Region | 81 | 22.8 | 593 | 24.5 | 0.499 |
| Pacific Region | 45 | 12.7 | 234 | 9.7 | 0.077 |
| Eastern-Orinoquia-Amazonia Region | 17 | 4.8 | 114 | 4.7 | 0.943 |
| Contributory regime | 257 | 72.4 | 1896 | 78.2 | 0.014 |
| Triptan type | - | - | - | - | - |
| Sumatriptan | 129 | 36.3 | 956 | 39.4 | 0.263 |
| Naratriptan | 124 | 34.9 | 786 | 32.4 | 0.348 |
| Sumatriptan/naproxen | 98 | 27.6 | 654 | 27.0 | 0.804 |
| Eletriptan | 4 | 1.1 | 28 | 1.2 | 0.963 |

**Supplementary Table 3**. Comparison of variables between participants with and without criteria for Medication-Overuse Headache.

| **Variables** | **MOH (Yes)** | | **MOH (No)** | | p |
| --- | --- | --- | --- | --- | --- |
|  | **n=19** | **%** | **n=326** | **%** |  |
| Women | 24 | 82.8 | 281 | 86.2 | 0.610 |
| Age, median (interquartile range) | 52.0 (42.0-60.5) | | 38.0 (29.0-50.0) | | <0.001 |
| Origin | - | - | - | - | - |
| Caribbean Region | 7 | 24.1 | 117 | 35.9 | 0.203 |
| Bogotá-Cundinamarca Region | 10 | 34.5 | 78 | 23.9 | 0.207 |
| Central Region | 9 | 31.0 | 72 | 22.1 | 0.271 |
| Pacific Region | 3 | 10.3 | 42 | 12.9 | 1.000 |
| Eastern-Orinoquia-Amazonia Region | 0 | 0.0 | 17 | 5.2 | 0.380 |
| Contributory regime | 26 | 89.7 | 231 | 70.9 | 0.030 |
| Migraine without aura | 16 | 55.2 | 192 | 58.9 | 0.696 |
| Severe headache attack | 28 | 96.6 | 290 | 89.0 | 0.200 |
| History of migraine ≥10 years | 22 | 75.9 | 129 | 39.6 | <0.001 |
| Comorbidities | - | - | - | - | - |
| Endocrine | 17 | 58.6 | 110 | 33.7 | 0.007 |
| Cardiovascular | 14 | 48.3 | 91 | 27.9 | 0.021 |
| Digestive | 8 | 27.6 | 51 | 15.6 | 0.098 |
| Psychiatric | 12 | 41.9 | 43 | 13.2 | <0.001 |
| Rheumatological | 11 | 37.9 | 41 | 12.6 | <0.001 |
| Neurological | 5 | 17.2 | 26 | 8.0 | 0.090 |
| Triptan type | - | - | - | - | - |
| Sumatriptan | 11 | 37.9 | 118 | 36.2 | 0.852 |
| Naratriptan | 12 | 41.4 | 112 | 34.4 | 0.447 |
| Sumatriptan/naproxen | 6 | 20.7 | 92 | 28.2 | 0.385 |
| Eletriptan | 0 | 0.0 | 4 | 1.2 | 0.549 |
| Correct dosage | 18 | 62.1 | 248 | 76.1 | 0.095 |
| Absence of pain after 24 hours -always or almost always- | 16 | 55.2 | 162 | 49.7 | 0.572 |
| Any adverse event | 6 | 20.7 | 140 | 42.9 | 0.020 |
| Previous use of simple analgesics | 20 | 69.0 | 139 | 42.6 | 0.006 |
| Conventional prophylaxis | 25 | 86.2 | 177 | 53.4 | 0.001 |
