# Supplementary figures and images for: Migraine and the Excessive Dispensation of Triptans: A Real-World Evidence Study of Colombian Patients
Source: Rev Neurol. 2026 Jan 26;81(1):46355. doi: 10.31083/RN46355 (PMC12873677; doi:10.31083/RN46355)

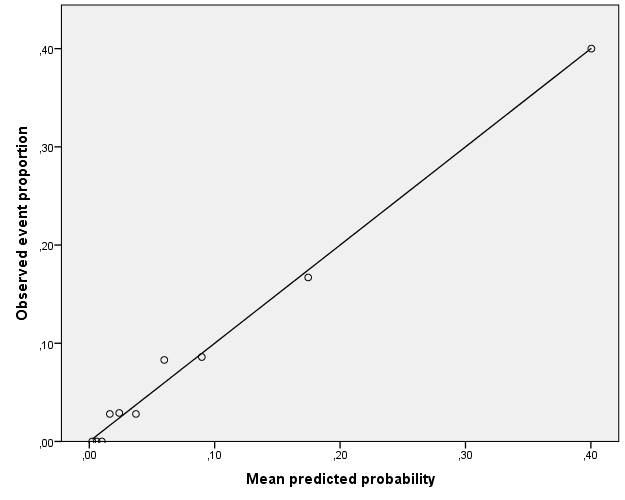

Supplement: Supplementary file 1 [file 1576-6578-81-1-46355-s1.zip › Supplementary Fig. 1 Calibration plot of the logistic regression with predicted probabilities and observed proportions of variables a.png]

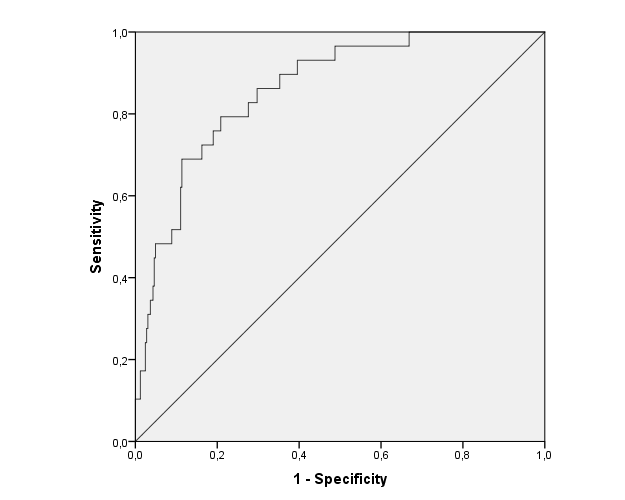

Supplement: Supplementary file 1 [file 1576-6578-81-1-46355-s1.zip › Supplementary Fig. 2 Receiver operating characteristic (ROC).png]
